# Remote Sensing Image Classification with a Graph-Based Pre-Trained Neighborhood Spatial Relationship

**DOI:** 10.3390/s21165602

**Published:** 2021-08-20

**Authors:** Xudong Guan, Chong Huang, Juan Yang, Ainong Li

**Affiliations:** 1 Research Center for Digital Mountain and Remote Sensing Application, Institute of Mountain Hazards and Environment, Chinese Academy of Sciences, Chengdu 610041, China; guanxd@imde.ac.cn; 2 State Key Laboratory of Resources and Environmental Information System, Institute of Geographic Sciences and Natural Resources Research, Chinese Academy of Sciences, Beijing 100101, China; huangch@lreis.ac.cn; 3 Shaanxi Energy Institute, Xianyang 712000, China; sxnyyj2014@163.com

**Keywords:** remote sensing, image classification, SVM (Support Vector Machine), knowledge graph, object-based image analysis, fuzzy classification, graph theory

## Abstract

Previous knowledge of the possible spatial relationships between land cover types is one factor that makes remote sensing image classification “smarter”. In recent years, knowledge graphs, which are based on a graph data structure, have been studied in the community of remote sensing for their ability to build extensible relationships between geographic entities. This paper implements a classification scheme considering the neighborhood relationship of land cover by extracting information from a graph. First, a graph representing the spatial relationships of land cover types was built based on an existing land cover map. Empirical probability distributions of the spatial relationships were then extracted using this graph. Second, an image was classified based on an object-based fuzzy classifier. Finally, the membership of objects and the attributes of their neighborhood objects were joined to decide the final classes. Two experiments were implemented. Overall accuracy of the two experiments increased by 5.2% and 0.6%, showing that this method has the ability to correct misclassified patches using the spatial relationship between geo-entities. However, two issues must be considered when applying spatial relationships to image classification. The first is the “siphonic effect” produced by neighborhood patches. Second, the use of global spatial relationships derived from a pre-trained graph loses local spatial relationship in-formation to some degree.

## 1. Introduction

Machine learning has been widely used in remote sensing image classification. Some studies have experimented on small regions, reaching an overall accuracy of more than 90% [1,2,3,4,5]. The accuracy is high enough at the national level if the overall accuracy is more than 85% [6]. In reality, however, there is still a large amount of remote sensing image classification requiring human interpretation or modification [6]. Traditional machine learning focuses on classification based on isolated information such as spectral, shape, and texture information for the extraction of ground features. Many researchers have flocked into the field of deep learning to seek breakthroughs in remote sensing image classification methods, precisely because traditional machine learning methods have shown bottlenecks that are difficult to break through [7,8,9,10,11]. For example, most machine learning methods are developed to converge upon a fixed solution, however, an ideal learning method should capable of continual learning by incorporate some common-sense knowledge. Deep learning has higher requirements on the number of training images, although transfer learning enables the network to adjust parameters and reduce the number of training images. The diversity of remote sensing imagery and the ground it depicts, however, make classification an extremely complex process, especially with the constant emergence of new sensors. Training for each sensor’s data and each geographic scene for remote sensing image classification is an unusually tough job for deep learning applications [12]. The knowledge of spatial relations within and between objects can be used as important knowledge in classification [13], because although there are many variables regarding sensor characteristics such as band ranges, spatial resolution, and revisit cycles, the distribution of the ground features usually has a certain pattern.

Early in 1987, scholars designed an expert system for remote sensing [14], a knowledge representation structure, and the knowledge contained in the system. Later, expert knowledge based on fuzzy logic was used to describe knowledge on the basis of the probability or degree [15]. There are also expert systems designed for land cover classification [16]. Early expert system knowledge representation for remote sensing imagery was usually based on a tree-like structure, which is conducive to rule-based inference such as If-Then rules [17], Dempster’s combination rule [18], or decision trees [19]. These expert systems usually contain information on spatial relationships between land cover types. It was difficult to apply that knowledge to classification until the emergence of object-based image analysis [20]. Object-based image analysis can easily include contextual and neighborhood information into the classification process [21]. Mutual relations between image objects include the similarity/dissimilarity of spectra between neighborhood objects, while contextual information includes spectral relationships to sub- and super-objects and spatial relationships such as ‘existence of,’ ‘border to’, and ‘distance to’ [22,23,24]. Qiao et al. [25] proposed a maximum spatial adjacency and directional spatial adjacency method to extract certain land cover classes. A representative platform, eCognition Developer, provides tools to perform a hierarchical rule-based classification scheme where the relationship between objects can be manually defined [26]. The object-oriented classification method has provided a solution to understanding image semantics [27].

Two issues are usually considered for knowledge-based classification of remote sensing images. First is to build a knowledge base. The key issue in building an appropriate knowledge base is to identify an appropriate data structure and knowledge representation (knowledge acquisition, conceptualization, and formalization [28,29]). Second is the implementation of knowledge. The key issue in implementing knowledge is to effectively use prior knowledge for interpretation and “scene understanding” for image classification. There are many recent studies focusing on knowledge-based methods for remote sensing data classification. Forestier et al. used a knowledge base constructed by ontology for scene and concept matching [29]. Rejichi et al. [30] designed an expert knowledge scheme for SITS (satellite image timeseries) analysis using scene ontology and a tree-like organized data structure. To localize the knowledge base, they proposed to compare similarity between an extracted graph from a knowledge base with the user request. Belgiu et al. [31] used existing literature on evaluated building types to build an ontology based on If-Then rules, random forest was used to classify the building types by the features described in the ontology. Forestier et al. [32] also used an object-oriented method for the classification of coastal areas, and an ontology describing existing classes in the region was built. A hypothesis about the semantics of the region was made and knowledge on the type of region was used to check and modify the hypothesis. Belgiu et al. developed a method to automatically embed a formalized ontology into an object-based image analysis (OBIA) process [33]. Objects produced by image segmentation are usually used as the basic processing units in the knowledge-based classification methods [34,35]. That is, knowledge is extracted by relationships between image objects rather than pixels. Recent years, ontology-based data structure is usually used for geographic knowledge representation.

To extract the knowledge of spatial relationships, Dale et al. reviewed and analyzed the value of geo-spatial graphs [36]. The authors suggested that “future applications should include explicit spatial elements for landscape studies of ecological, genetic, and epidemiological phenomena”. Cheuang et al. [37] practiced a graph-based representation of landscape relationships where a graph-based structure was implemented to present landscape topology. Moreover, graph edit distance was leveraged to project the structural attributes of a landscape entity’s topology to vector dimensions. The graph-based data structure enables more analysis regarding spatial relationships between landscape entities such as subgraph mining and kernel analysis. Recently, Xu et al. [38] used graphs to explore the morphological changes between adjacent tidal flat objects. Aside from directly using graph-based spatial relationships in the process of classification, graph convolutional neural networks (GCN) have been implemented in remote sensing image classification. Ouyang and Li proposed a method to first extract features of objects and then constructed a graph containing the extracted features of objects for the implementation of a GCN classifier [39]. Li et al. proposed a scene classification scheme that first extracted scene features then segmented the feature map to construct a graph. Finally, the graph was classified by a graph attention network (GAT) [40]. Ma et al. proposed a sum of minimum distance parameter to determine graph adjacency relationships. The parameter was used for the classification of hyperspectral image (HSI) data [41]. Pu et al. reviewed the application of GCN on HSI classification and proposed a graph-based CNN (Convolutional Neural Network) classifier to classify HSI data [42]. Scholars in the remote sensing community usually leverage graphs to extract spatial relationships.

Motivated by the notion that the graph-based structure can learn adjacency information, we propose a method to make use of adjacency information by combining information at the decision level. The proposed method consists of three parts. First, the original image is segmented and classified by a fuzzy classifier to produce a membership, which represents the probability they belong to a class. In the second step, a graph based on an existing land cover map is produced to calculate the probability of adjacency between land cover types. In the final step, the two probabilities are assembled to produce the final class decision. The main contributions of this paper can be summarized as follows:

- We propose a method to extract the adjacency probability by using a graph.
- The adjacency probability derived from the graph is aggregated with pre-classification results at the decision level.
- Two experiments show that the method has some ability to correct misclassified objects with neighborhood information, but problems regarding global uncertainty and the “siphonic effect” need to be considered in future work.

The remainder of this paper is organized as follows. Section 2 introduces the details of our proposed framework. Section 3 describes the setup of the experiments and reports the results. Section 4 discusses the factors of our framework. Section 5 presents the conclusions.

## 2. Method

**Figure 1:**
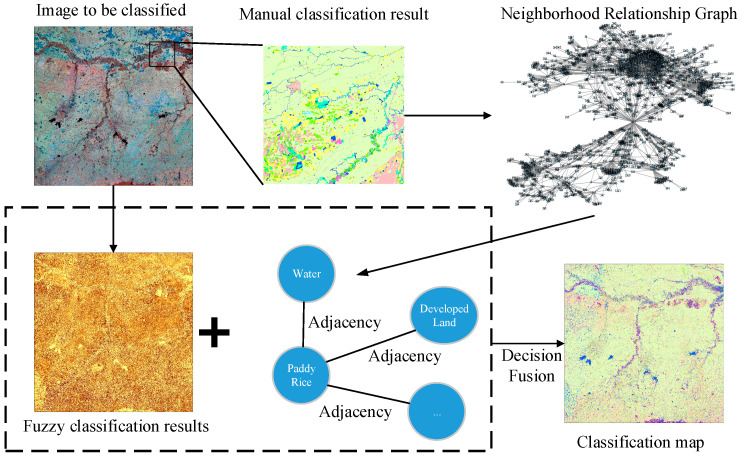
Overview of the proposed framework.

Our proposed framework is illustrated in Figure 1. Generally, we proposed a way to train previous knowledge of the distribution pattern of land cover types using graph theory and aggregated the trained distribution pattern with physical features of the land cover to the final decision module. Specifically, because manually interpreting and classifying small regions enabled us to get an accurate spatial distribution pattern of land cover types, we trained the previous knowledge by manually interpreting and classifying small regions as ROI (Region of Interests) of the region to be classified. Second, we built a graph of the land cover features of the ROI and calculated each node’s degree by land cover type. This degree represents the probability of the presence of neighboring land cover types. Third, fuzzy classification was conducted on the image to obtain the membership. Finally, we combined the probability of the presence of neighboring land cover types with membership to reach the final decision.

### 2.1. Generating the Spatial Relationship Graph

To generate pre-trained knowledge on the relationships between classes, a simple graph-based method was used. First, a small region was chosen as an ROI within the area to be classified, and the area was manually interpreted and classified to produce a land cover map. Manual interpretation is usually used as reference map because of its accuracy This can also be done by classifying very-high resolution remote sensing images [43,44], but the classified land cover map must be processed using a generalization technique to guarantee the integration of converted features. Small features should be merged into neighboring polygons. Because of landscape may changes overtime hence effects adjacency possibility. We suggest that using images collected near the to-be-classified image dates for manually interpretation.

**Figure 2:**
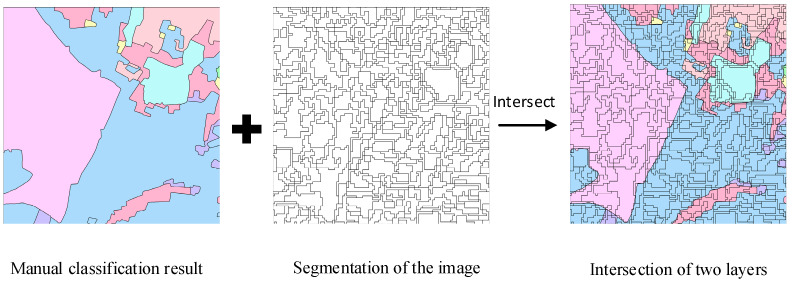
Process to obtain the feature map for graph construction.

Second, the image was segmented using the same segmentation scales as the whole region. The segmented image was intersected with the manually classified land cover map to guarantee the object adjacent to each node had the same scale with the image to be classified. For example, in the manually classified land cover map, land cover areas of the same type are outlined as one feature, but when segmenting the image to be classified, adjacent land cover areas of the same type may be segmented as separate objects. The manually classified land cover map has few adjacent features with identical types, but the segmented map has many. Intersection of the segmented features with the land cover map can reflect an identical segment scale with the image to be classified, showing the probability of adjacent features. The process is shown in Figure 2.

The polygons of the segmented manually classified land cover map are denoted as a set *SMC*. Assume there are k polygons in SMC, and SMC= {x |0<x≤k, x∈Z)}. Each polygon x should have a class type c. Assume there are n types of land cover in the area.

Third, the graph G =(V, E) was built using the manually classified land cover map. Set V is a set of nodes in G, which are polygons x of the manually classified land cover map. Set E is a set of edges in G, which have no direction nor weight and denote the adjacency relationship between polygons. The objective was to get the probability of each type c adjacency to {c |0<c≤n, c∈Z)}. The probability of polygons with type $c_{i}$ adjacency to polygons with type $c_{j}$ were calculated via the graph G using the degree of nodes V. The calculation is shown in Equation (1):

(1) $$P_{cij}=\frac{\sum_{v\in c_{i},\;v\in c_{j}}e}{\sum_{v\in c_{i}}\deg\left(v\right)}$$

where $P_{cij}$ is the probability that type $c_{i}$ is adjacent to type $c_{j}$; deg(v) is the degree of node v; $\underset{v\in c_{i}}{\sum }\deg\left(v\right)$ is the degree of nodes v, which have the attributes that belongs to type $c_{i}$; and $\underset{v\in c_{i},\;v\in c_{j}}{\sum }e$ is the number of edges that connect nodes v, which have the attributes that belongs to type $c_{i}$, and nodes v which have the attributes that belongs to type $c_{j}$.

### 2.2. Object-Oriented Fuzzy Classification

An object-oriented method for pre-classification was used to reduce the data processing time. More importantly, the object-oriented method better reflects relationships between classes. The degree to adjacent land cover is more robust and enables the built graph to be more applicable and extensible to larger regions [45]. This is because images with different resolutions will show different probabilities of adjacency. Images with a higher resolution will need more pixels to store real surface features, which will make the probability of adjacency smaller.

It is labor-intensive to produce a land cover map of a sample region, though reference land cover maps from high-resolution satellite imagery have a high accuracy of classification. To transform the high-resolution classification data, the strategy in this paper is to overlay the segmented polygons on the high-resolution land cover map and assign major land cover types within each polygon as the land cover map of the segmented polygon.

#### 2.2.1. Image Segmentation

The eCognition platform uses five parameters to control multi-resolution segmentation (MRS), including the scale, shape, color, compactness, and smoothness parameters. The segmentation scale is the most critical parameter that controls the size of resultant polygons. A good segmentation will produce a balance between polygon size and the homogeneity within an object and heterogeneity between objects [46,47]. The shape and color parameters define the weight that the shape and color criteria should have when segmenting the image. The higher the value of the shape, the lower the influence of color on the segmentation process. For the compactness and smoothness criteria, the higher the weight value, the more compact image objects may be. Note that different test sites should have different segmentation parameter values.

#### 2.2.2. Selected Fuzzy Classifier

1. Nearest neighbor

Nearest neighbor (NN) classification first builds a feature space using spectrum, geometry, or texture of samples [48]. Then each object is classified by mapping its features to the feature space. Finally, the Euclidean distance between sample features is used with the object’s features for classification. The NN classification is based on the minimum distance in the NN feature space where the training data are constructed by spectral, shape, or texture feature values. The distance can also be seen as the reliability of the classification results. The distance function is shown in Equation (6) [49]:

(2) $$d\left(x,y\right)={\left(\overset{m}{\underset{i=1}{\sum }}{\left(x_{i}-y_{i}\right)}^{2}\right)}^{1/2}$$

where d(x,y) is the Euclidean distance of samples to be classified in the NN feature space. The data are more similar to the samples when the Euclidean distance is smaller. The Euclidean distances provide a chance to range the feature values into fuzzy membership values between 0 and 1.

After the segmentation, the objects were classified via sample points. The fuzzy classification process was also performed in eCognition. The object information, including the spectral, texture, shape, and difference with neighbor objects were input in the NN feature space for the training of samples. The distance was output as membership to use in the decision phase.

1. Fuzzy SVM

SVM is another popular classification technique [50]. The principle of SVM can be briefly described as follows [51]. Given a set of instance-label pairs ($x_{i},\;y_{i}$), i=1, …, l  where $x_{i}\in R^{n}$ and $y_{i}\in {\left\{1,-1\right\}}^{l}$, the SVM requires the solution of the following optimization problem:

$$\underset{w,b,\xi }{\min}\frac{1}{2}w^{T}w+C\overset{l}{\underset{i=1}{\sum }}\xi_{i}$$

(3) $$\mathrm{Subject}\;\mathrm{to}\;y_{i}\left(w^{T}\phi \left(x_{i}\right)+b\right)\geq 1-\xi_{i},$$

$$\xi_{i}\geq 0$$

Here, training vectors $x_{i}$ are mapped into a higher (maybe infinite) dimensional space by the function ϕ. The SVM finds a linear separating hyperplane with the maximal margin in this higher dimensional space. C>0 is the penalty parameter of the error term. Furthermore, $K\left(x_{i},\;x_{j}\right)=\phi {\left(x_{i}\right)}^{T}\;\phi \left(x_{j}\right)$ is called the kernel function. The most commonly used kernel function is the Gaussian radial basis function (RBF):

(4) $$K\left(x_{i},\;x_{j}\right)=\exp\left\{-\frac{{\left\|x_{i}-x_{j}\right\|}^{2}}{2\sigma^{2}}\right\}$$

Fuzzy SVM means the output is a probabilistic prediction. Hong and Hwang [52] provided a strategy in training the SVM and mapping the outputs into probabilities. The probability is measured by Bayesian theory, and the kernel model is replaced by a Bayes formula:

(5) $$P\left(y=1|\;f\right)=\frac{1}{\exp\left(Af+B\right)}$$

where y∈{−1,1} is the label and f is the decision function:

(6) $$f\left(x\right)=\underset{i\in S}{\sum }\alpha_{i}y_{i}K\left(x_{i},\;x\right)+b$$

where {$\alpha_{i}$} is a set of nonzero multipliers. For multiple classes, Equation (7) is used:

(7) $$\underset{p}{\min}\frac{1}{2}\overset{k}{\underset{i=1}{\sum }}\underset{j:j\neq i}{\sum }{\left(r_{ji}p_{i}-r_{ij}p_{j}\right)}^{2}\;\overset{k}{\underset{i=1}{\sum }}p_{i}=1$$

where $r_{ij}$ is the probability that the sample belongs to class i when considering only the two classes i and j so that the one-one classification is transferred into a one-all classification.

### 2.3. Aggregation of Graph and Membership

We obtained the membership of each object according to Section 2.2 as well as the probability of land cover adjacency. The membership of polygons can be treated as the probability that the polygon belongs to a land cover type. The classification is based on spectral, shape, or texture information of objects and does not contain neighborhood information of objects. The degree obtained from the neighborhood relationship graph contains the probability of land cover types that are adjacent to other types. We can combine the two probability values by a decision fusion scheme:

(8) $$C=P_{membership}\times P_{neighborhood}$$

where C is the classification result of the polygon; $P_{membership}$ is the probability obtained by the fuzzy classification based on spectral, shape, and texture information of the object; and $P_{neighborhood}$ is the probability obtained by neighborhood information from the pre-trained graph.

More specifically, each node contains the fuzzy classification membership of each polygon. Also, we obtained the probability that the land cover is adjacent to others. Supposing that a polygon has n adjacent polygons, the adjacent polygons of polygon *Y* were denoted as $\left(N_{X}|X=1,\;2,\;\ldots n\right)$. Supposing there are k types of land cover, the land cover types were denoted as $T=\{t\;|\;t=t_{1},\;t_{2},\;\ldots t_{k}\}$. The membership of polygons are given as $M=\{m_{t}\;|\;t=1,\;2,\;\ldots k\}$ and the probability of land cover $t_{i}$ adjacent to $t_{j}$ is $P_{t_{i},t_{j}}$. The fusion was conducted as the following equation:

(9) $$N_{Ym_{i}}=\overset{n}{\underset{X=1}{\prod }}\overset{k}{\underset{j=1}{\sum }}N_{Ym_{i}}\times N_{Xm_{j}}\times P_{t_{i},t_{j}}$$

where $N_{Ym_{i}}$ is the membership value of type i in polygon *Y*; *X* is the adjacent polygons to polygon *Y*; $N_{Xm_{j}}$ is the membership value of type j in polygon *X*; and $P_{t_{i},t_{j}}$ is the probability of land cover $t_{i}$ adjacent to $t_{j}$ obtained by the built graph in Section 2.1.

The final decision of land cover type in polygon *Y* is max ($N_{Ym_{i}}\;|\;i=1,\;2,\;\ldots k)$. The aggregation equation is intended to consider the land cover types of the neighborhood of the polygon to be classified as well as the fuzzy classification result on its own.

## 3. Experiments

The purpose of using a graph is to make use of previous knowledge on the distribution of land cover types. Our scheme used a localized knowledge of the probability of land cover adjacency and a simple degree of the nodes. The information of this localized knowledge was used by the decision fusion of membership and the probability of land cover adjacency.

### 3.1. Study Area and Satellite Data

#### 3.1.1. Brief Introduction to the Study Area

We used two satellite images to test on two test sites. One region is the Mun River Basin, the other is Kent County, Delaware, USA. The land cover of the Mun River Basin is mainly paddy rice, while Kent County consists of multiple land cover classes such as forest, agriculture, and cities.

1. Mun River Basin

**Figure 3:**
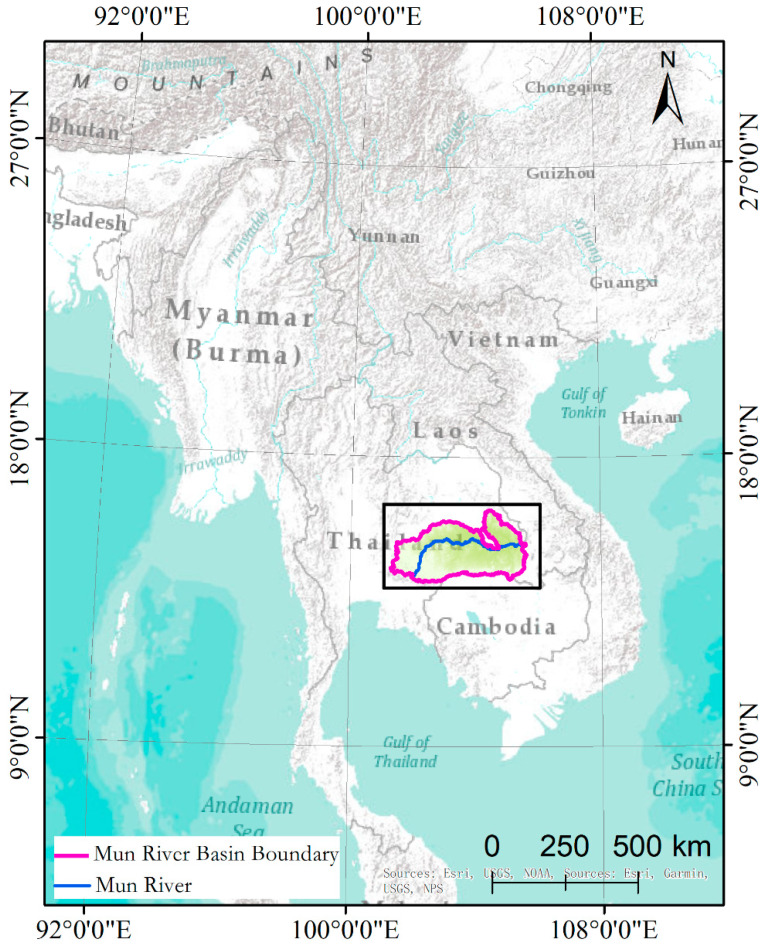
Location of the Mun River Basin test site in northeastern Thailand.

The Mun River Basin is located in northeastern Thailand bordering Laos to the east and Cambodia to the south, between 101°30′–105°30′ E and 14°–16° N. It is the largest river basin in Thailand, the largest river on the Khorat Plateau, and the second longest river in Thailand (the largest is the Chak River). It is also a major tributary of the Mekong River. The main stream of the Mun River is about 673 km long and the basin area is about 70,500 km^2^. Vegetation coverage in the Mun River Basin is large, about 12% natural vegetation and about 80% artificial vegetation, and the rest of the land cover types are water bodies and developed land. The location of the area is shown in Figure 3.

2. Kent County, Delaware, USA

**Figure 4:**
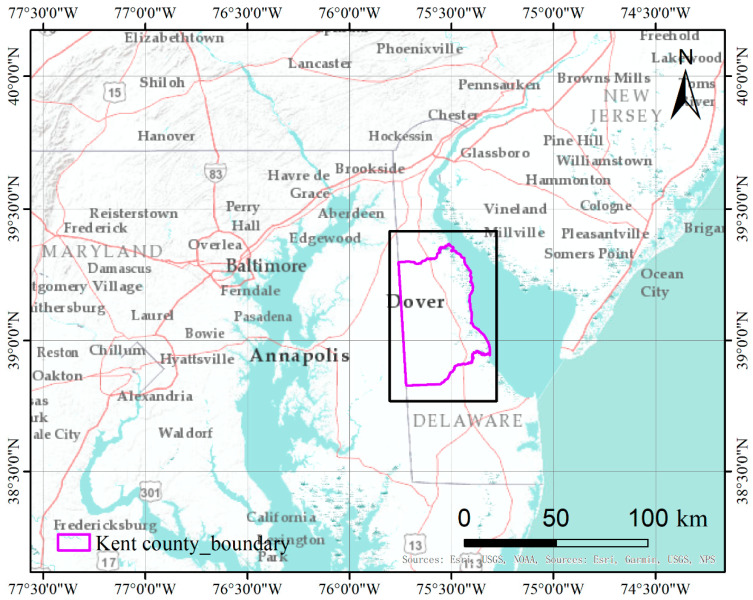
Location of the Kent County test site in the Chesapeake Bay area.

Kent County is located in the central part of the U.S. state of Delaware within the Chesapeake Bay area, the largest estuary in the United States [53]. Kent County has a humid subtropical climate according to the Köppen climate classification, while the Trewartha climate classification considers the climate oceanic because only seven months average >10° C (>50° F). All months average above freezing and Dover has three months averaging above 22° C (71.6° F.) The hardiness zone is mostly 7a with very small areas of 7b [54]. The location of the site is shown in Figure 4.

#### 3.1.2. Satellite Data and Pre-Processing

1. Landsat 8 OLI

Landsat 8 data from the United States Geological Survey (USGS) website (https://earthexplorer.usgs.gov/, accessed on 15 August 2021) were used for the Mun River Basin, selecting high-quality images (cloud cover less than 10%) from 2015. The Landsat 8 satellite carries the Operational Land Imager (OLI) including nine bands, among which eight are multispectral bands with a resolution of 30 m and another 15 m panchromatic band. The imaging width is 185 km × 185 km. The Landsat 8 OLI image used is a Level 1T product. We conducted radiometric calibration and FLAASH model-based atmospheric correction with the ENVI 5.0 SP3 software. Then the Landsat 8 OLI data were resampled to 25 m with the nearest resampling technique.

To obtain an accurate land cover map by manual interpretation and classification, Google Earth imagery was downloaded and georeferenced using Landsat data and DEM images (Shuttle Radar Topography Mission, SRTM, 30 m data).

2. Sentinel-2

Sentinel-2 data were used in Kent County, Delaware, downloaded from the CREODIAS website (https://creodias.eu/, accessed on 15 August 2021). Sentinel-2 is a wide-swath, high-resolution, multi-spectral imaging mission, supporting Copernicus Land Monitoring studies, including the monitoring of vegetation, soil, and water cover, as well as observation of inland waterways and coastal areas. It uses a Multispectral Instrument (MSI) that samples thirteen spectral bands: four bands at 10 m, six bands at 20 m, and three bands at 60 m spatial resolution. The acquired data, mission coverage, and high revisit frequency provide for the generation of geoinformation at local, regional, national, and international scales. We used high-quality images (cloud free) from 2016. The downloaded level 1 data were processed with orthographic correction and geometric correction on a sub-pixel level. Atmospheric correction should be conducted in principle, but the quality of the data was good enough for classification and we did not conduct atmospheric correction. We only used four bands at 10 m for classification, including the Red, Green, Blue, and NIR bands.

### 3.2. Results of the Mun River Basin

We first built the pre-trained graph for spatial relationship extraction by selecting regions that contain all types of land cover to perform manual interpretation and classification.

#### 3.2.1. Trained Graph in the Mun River Basin

The sampling region’s area of 672 km^2^ comprised 3710 polygons in the manually classified land cover map. After the segmentation and intersection, 76,141 polygons were generated. For the graph, 498,908 edges were generated for the 76,141 nodes. The edges denote that the nodes are adjacent to each other. We visualized 9722 nodes and their 63,432 edges.

From the graph, statistics were calculated for each node’s degree with different labels according to Equation (1). For example, nodes labeled “artificial forest” have 4000 degrees. Among the edges connected to nodes labeled “artificial forest”, 200 nodes are labeled “wetland”. The probability of “artificial forest” being adjacent to “wetland” is 200/4000. Table 1 shows the probability of land cover adjacent to other land cover types. Using this probability, we can aggregate the fuzzy classification result with the adjacent probabilities.

#### 3.2.2. Pre-Fuzzy-Classification Results in the Mun River Basin

**Figure 5:**
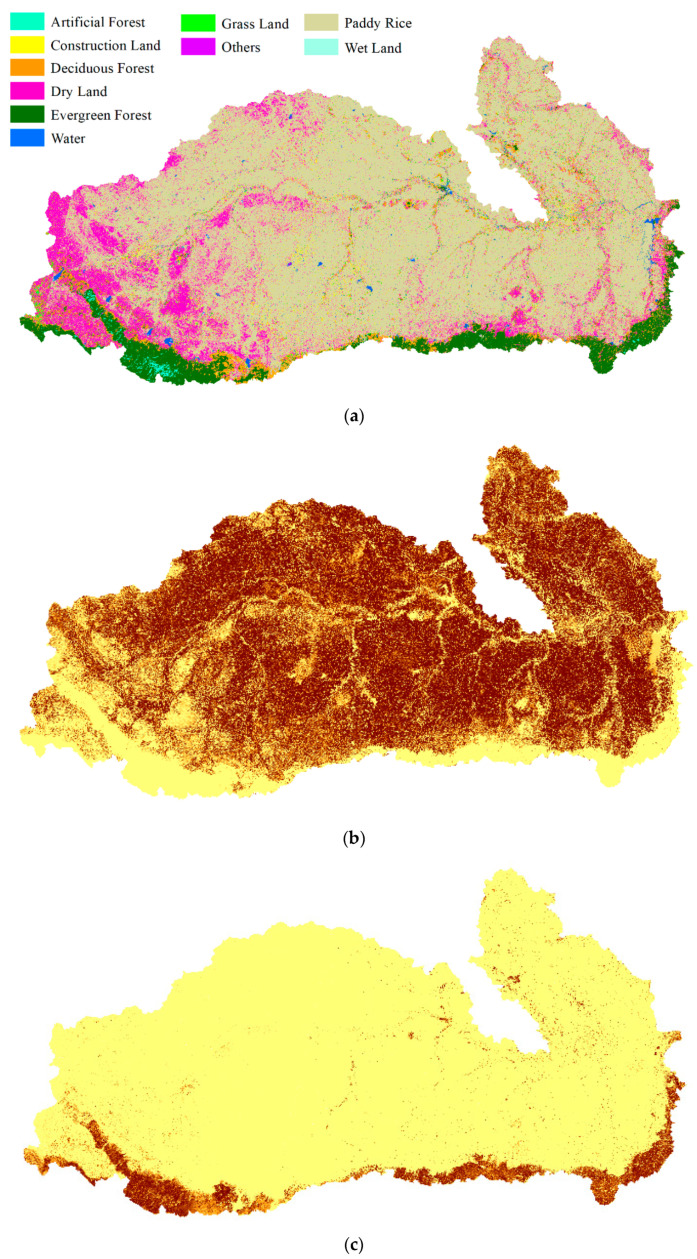

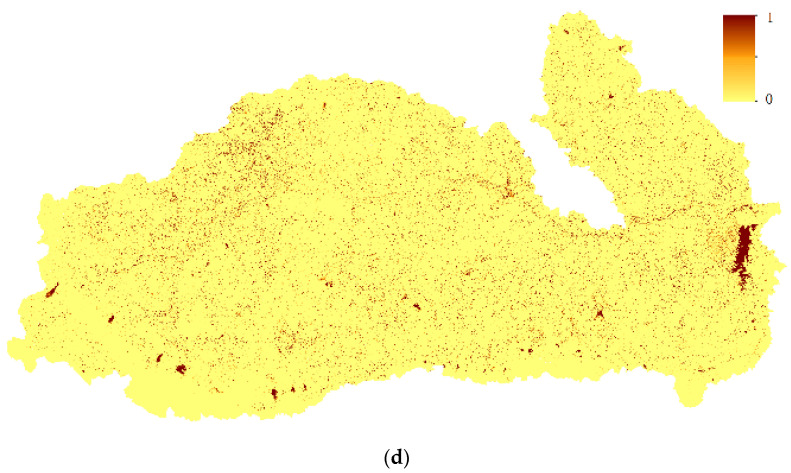
Fuzzy classification results and membership of selected land cover types in the Mun River Basin: (**a**) classification result by nearest neighbor; (**b**) membership of paddy rice; (**c**) membership of evergreen forest; (**d**) membership of water.

The nearest neighbor classifier used 6000 points for the classification of the Mun River Basin. We used a relatively large number of sample points for training to guarantee the accuracy of pre-classification results. Thus, the improvement in the fusion stage was not caused by the low accuracy of pre-classification results. Fuzzy classification using the nearest-neighbor classifier was implemented in eCognition with a scale parameter of 60 in the multi-resolution segmentation model, a shape criterion of 0.1, and a compactness of 0.5. All bands participated in the segmentation. In the classification process, we chose the mean value of each band, the NDVI value, and the width/length value to input into the nearest neighbor feature space. The result is shown in Figure 5a. Membership of selected land cover types including paddy rice, evergreen forest, and water is also shown in Figure 5.

#### 3.2.3. Decision Fusion Map of the Mun River Basin

**Figure 6:**
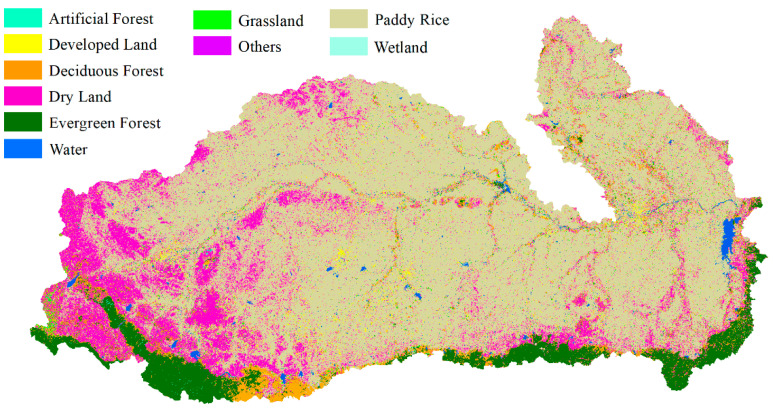
Land cover map of the aggregated pre-trained neighborhood spatial relationship and fuzzy classification membership.

According to Equation (9), the fuzzy classification results were aggregated with neighborhood land cover probability. A decision fusion map of the Mun River Basin is shown in Figure 6.

The major pattern of land cover distribution did not have many differences compared with the pre-classification map and true land cover classification map. The main land cover class is paddy rice. Dry land is mostly distributed in the west of the basin while water and developed land are distributed evenly. Forests, including deciduous and evergreen, are mostly distributed in the south.

#### 3.2.4. Accuracy Assessment of the Mun River Basin

**Figure 7:**
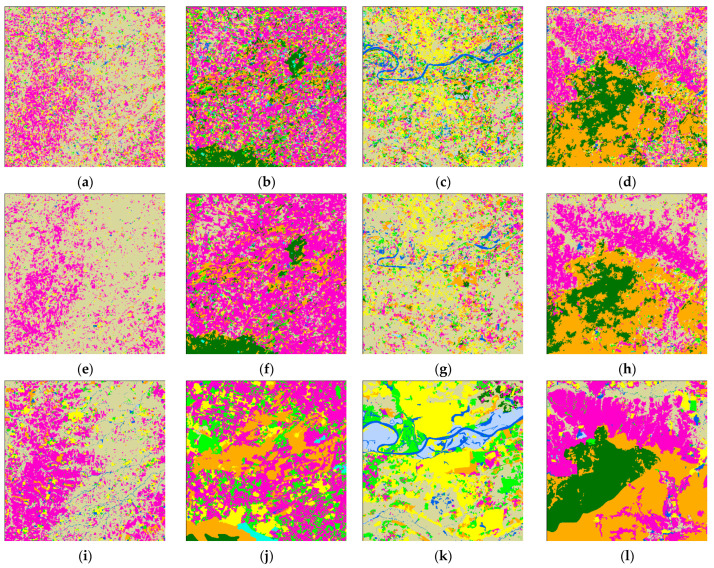

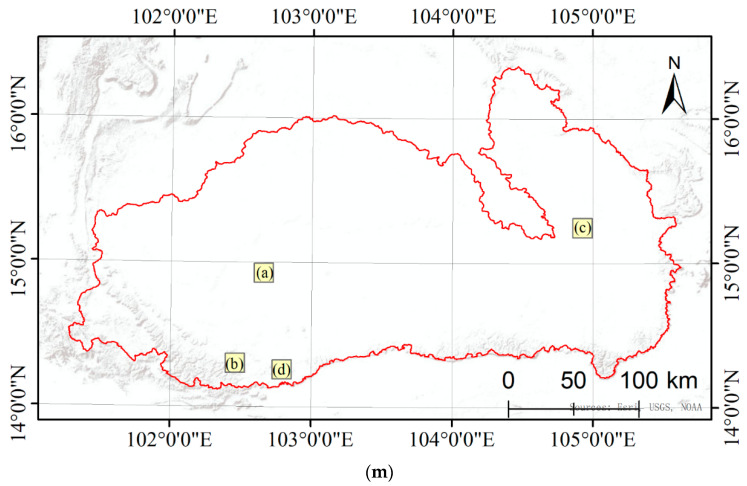
Details of the pre-classification land cover map versus the decision fusion land cover map. Four locations mainly containing paddy rice, dryland, developed land, and forests are shown at a larger scale: (**a**) mainly paddy rice, dry land, and developed land; (**b**) mainly dryland and forest; (**c**) mainly paddy rice, water, and developed land; (**d**) mainly dryland and forest; (**e**–**h**) the same regions with decision fusion results; (**i**–**l**) the same regions with manually interpreted land cover. (**m**) is the location of the four regions.

In order to compare the detailed differences between the pre-classification map, fusion map, and true land cover classification map, four regions were chosen and shown at a larger scale for demonstration. The main land cover types in each are, respectively, paddy rice, dry land, and developed land; dryland and forest; paddy rice, water, and developed land; and dryland and forest. Comparisons are shown in Figure 7.

As shown in Figure 7a,e,i, the pre-classification results preserved more details of dry land and developed land. Figure 7b,f,j show that the pre-classification map and decision map are not very similar to the true land cover map. On the fusion map, more patches are classified as dry land. The evergreen forest in the bottom left is more intact on the decision map than the pre-classification map. More dry land is classified in the fusion map compared with the pre-classification map. In Figure 7c,g,k, the most obvious difference in the decision fusion map and pre-classification map is that the decision fusion map failed to correctly classify the water in the middle of the river. In Figure 7d,h,l, the distribution of land cover does not have much of a difference with the pre-classification map. However, the decision fusion map captured more intact patches compared with the pre-classification map. These results demonstrate that fusion after aggregation loses details in the land cover map. Patches are more intact compared with the pre-classification map of the nearest neighbor classifier.

**Figure 8:**
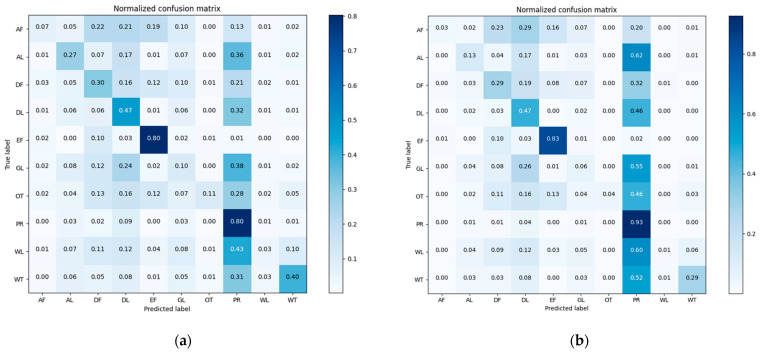
Confusion matrixes of the Mun River Basin for (**a**) pre-classification results and (**b**) neighborhood spatial relationship and fuzzy classification fusion results.

Confusion matrixes of the classification results of pre-classification and after-fusion are shown in Figure 8. We normalized the confusion matrix [55] for visual comparison.

**Figure 9:**
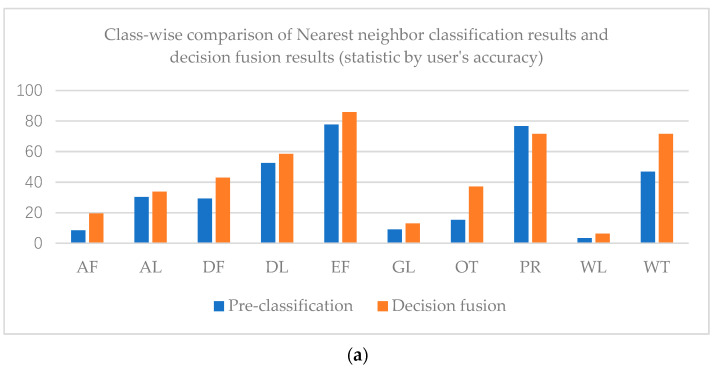

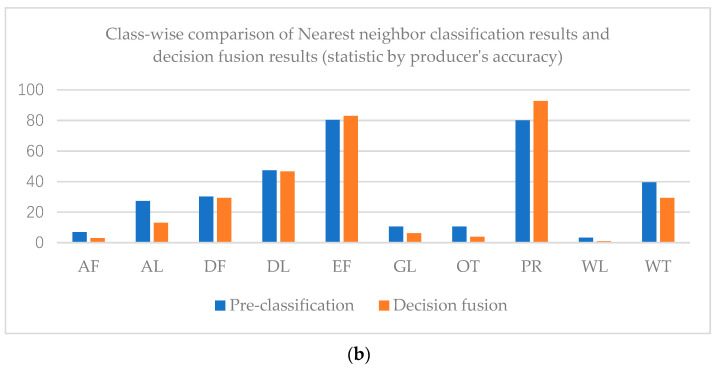
Class-wise comparison of nearest neighbor classification results and decision fusion results (statistic by user’s accuracy (**a**) and producer’s accuracy (**b**)) in the Mun River Basin.

The overall accuracy of the nearest neighbor classification result is 61.71%, while that of the decision fusion result is 66.95%. As to the Kappa coefficient, the nearest neighbor classification kappa coefficient is 0.42 and the decision fusion result is 0.43. Paddy rice’s predicted label increased in the decision map, as did evergreen forest, while other classes decreased. Class-wise comparison of the nearest neighbor classification results and decision fusion results are shown in Figure 9.

As shown in Figure 9, user’s accuracies of all classes in the decision fusion results are higher than the pre-classification results. However, only the evergreen forest and paddy rice producer’s accuracies of decision fusion results are higher than the pre-classification results.

### 3.3. Results for Kent County

Following the method above, we also conducted the experiment for Kent County.

#### 3.3.1. Trained Graph in Kent County

Unlike the Mun River Basin’s true land cover map, which was obtained by manually interpreting and classifying land cover, the “true” land cover map was obtained by a 1 m computer classification. To achieve a better effect on the scale issue we introduced in 2.2, the segmentation polygon was overlaid on the 1 m land cover map. Segmentation polygon is obtained by multi-resolution segmentation of the sentinel-2 image on the eCognition development platform. Segmentation scale is set as 80. And a shape criterion of 0.1, and a compactness of 0.5.

**Figure 10:**
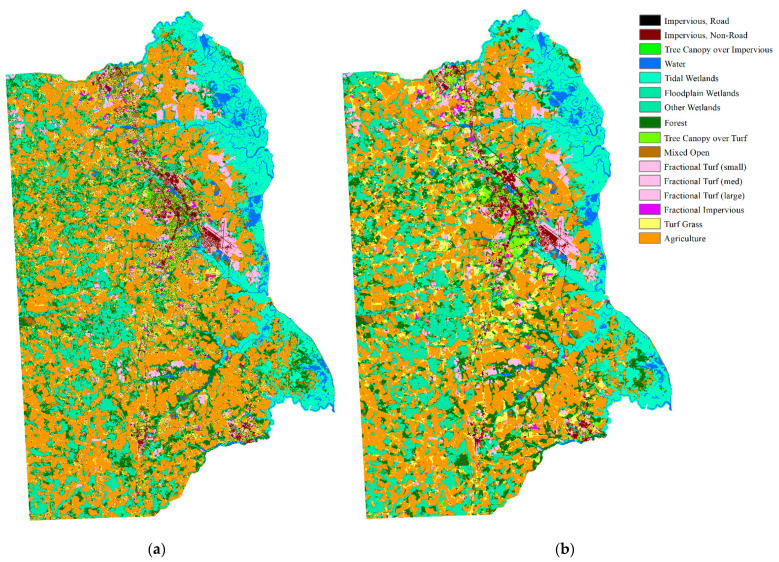
Comparison of 1 m land cover map and the overlaid segmentation map: (**a**) 1 m land cover map; (**b**) overlaid segmentation map.

We assumed that there was only one type of land cover within an object. Land cover types were accounted for within the segmented polygon of the land cover map and the land cover type with the highest count was treated as the land cover of the object. The comparison of the land cover maps is shown in Figure 10.

**Figure 11:**
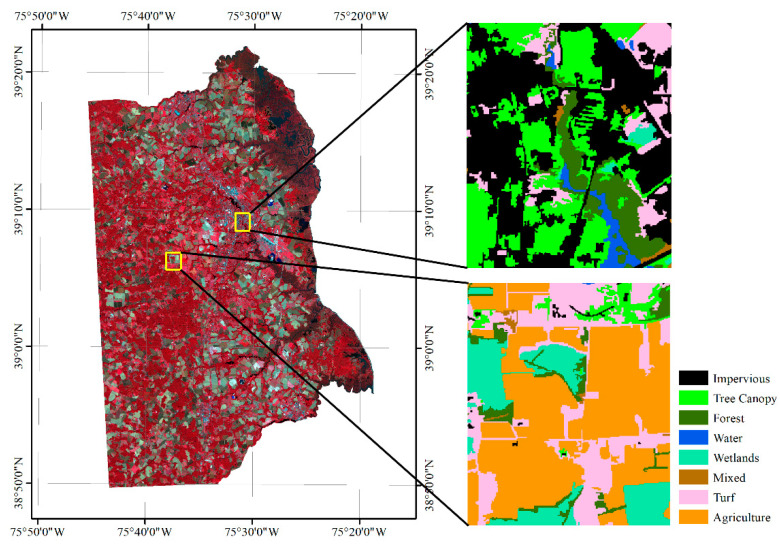
Location of the sample region for building the graph of Kent County.

Unlike the homogeneous landscape of the Mun River Basin, Kent County can roughly be divided into urban and suburban landscapes. Two small regions were selected to represent the urban region and suburban region to build the graph. The overlaid segmentation map is coarser and loses some details in the 1 m image classification map, but the overall land cover types are identical. In a real application, it is not necessary to transfer the entire high-resolution land cover map to the object level. Only sample regions that are used for graph building are needed (Figure 11).

**Figure 12:**
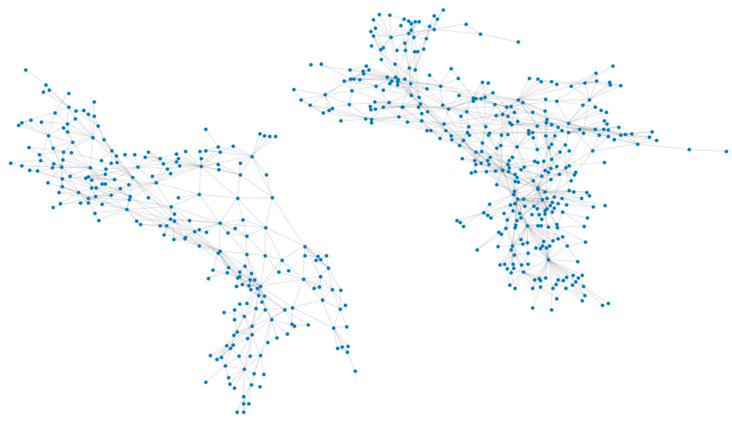
Visualized graph for the pre-trained spatial relationship of Kent County.

Because of the spectral similarity and the definition homogeneity, we combined some of the classes. The final classification scheme has eight types of land cover: impervious, tree canopy, water, wetlands, forest, mixed land, turf, and agriculture. In total, 2662 edges were generated for the 603 nodes for graph building. The edges denote that the nodes are adjacent to each other. We visualized the built graph of the two sample regions in Figure 12.

From the graph, the statistics of each node’s degree with different labels according to Equation (1) were calculated. The calculation of the probability of land cover types being adjacent to each other in Kent County were the same as the Mun River Basin. Table 2 shows the probability of land cover types being adjacent to other types. Using this probability, we can aggregate the fuzzy classification result with the adjacent probabilities.

#### 3.3.2. Pre-Fuzzy-Classification Results in Kent County

**Figure 13:**
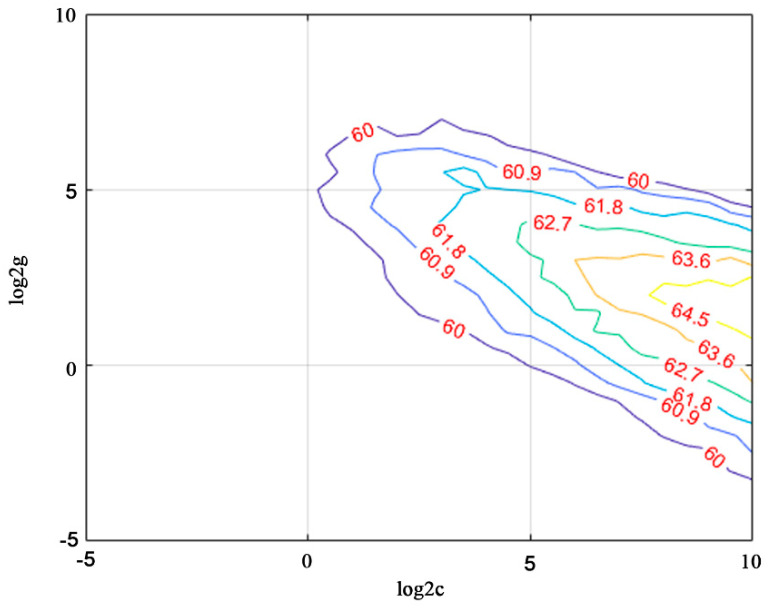
Training for c and g values for the RBF kernel of the SVM classifier.

LIBSVM 3.2.4 was implemented for pre-classification in Kent County [56]. C-SVC was implemented for the SVM model and RBF was run as the SVM kernel. Parameter values were trained on 1500 samples and five folds of cross-validation accuracy was 43.35%. The best c value is 724.0773 and the best g value is 2.8284, as shown in Figure 13. However, testing 6000 samples yielded an accuracy of 65.55% (statistics of object numbers rather than area). Therefore, we decided to use this parameter for SVM model training.

**Figure 14:**
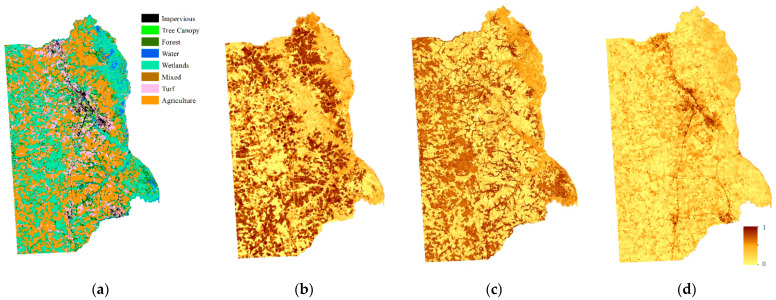
Fuzzy classification results and selected land cover type membership in Kent County: (**a**) classification result by SVM; (**b**) membership in agriculture; (**c**) membership in forest; (**d**) membership in impervious.

LIBSVM uses an All-versus-All strategy to achieve multi-label classification. We directly used the multi-classification solution for our classification. The pre-classification result in Kent County is as shown in Figure 14a. Membership in the selected land cover types including impervious, forest, and agriculture is also shown in Figure 14.

#### 3.3.3. Decision Fusion Maps in Kent County

**Figure 15:**
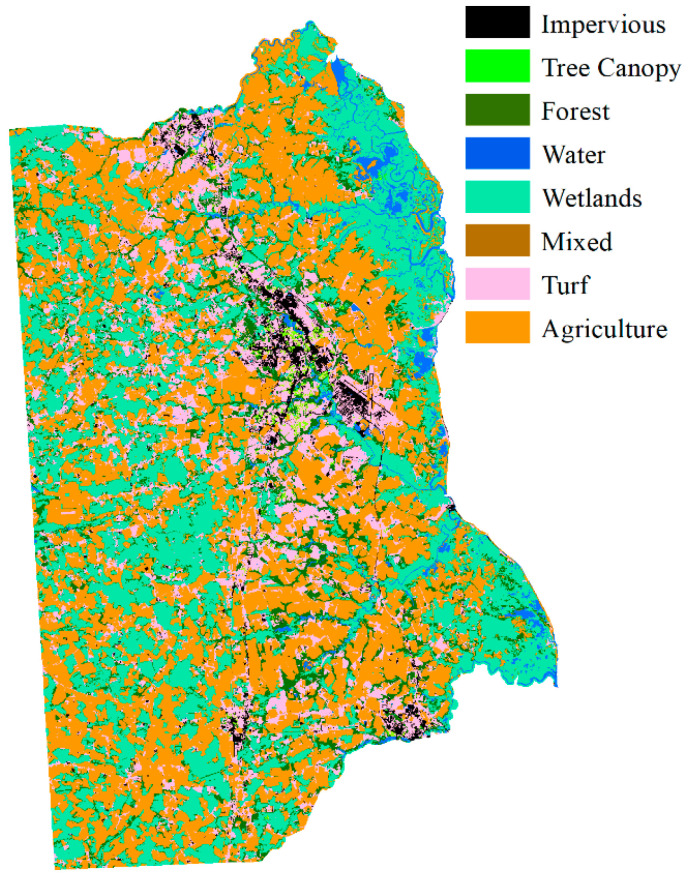
Land cover map of the aggregated pre-trained neighborhood spatial relationship and fuzzy classification membership result.

According to Equation (9), the fuzzy classification results were aggregated with neighborhood land cover probability. The decision fusion map of Kent County is shown in Figure 15.

#### 3.3.4. Accuracy Assessment of Kent County

In order to compare the detailed difference between the pre-classification map, fusion map, and true land cover classification map, three small regions were randomly picked to show the detailed difference between pre-classification results and decision fusion results. The three small regions mainly contained wetland and agriculture; impervious and tree canopy; and water and wetlands.

**Figure 16:**
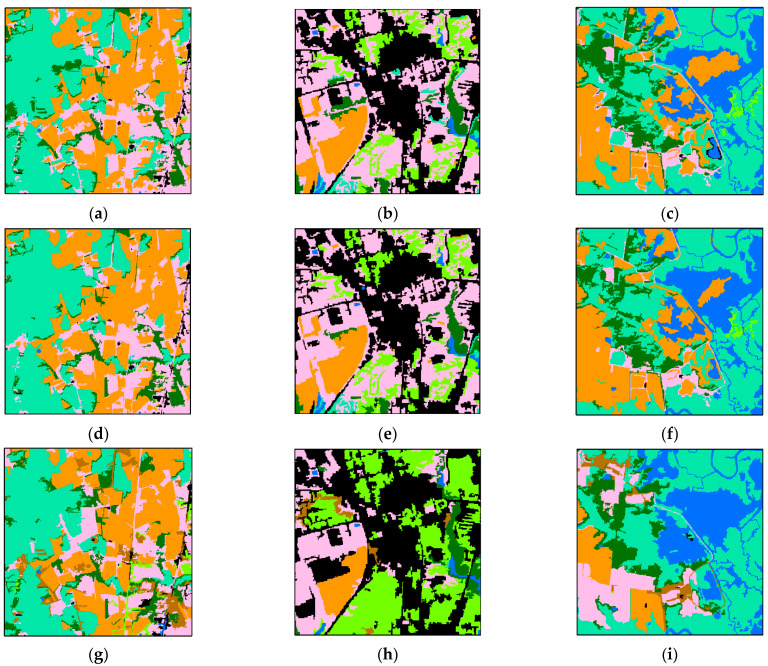

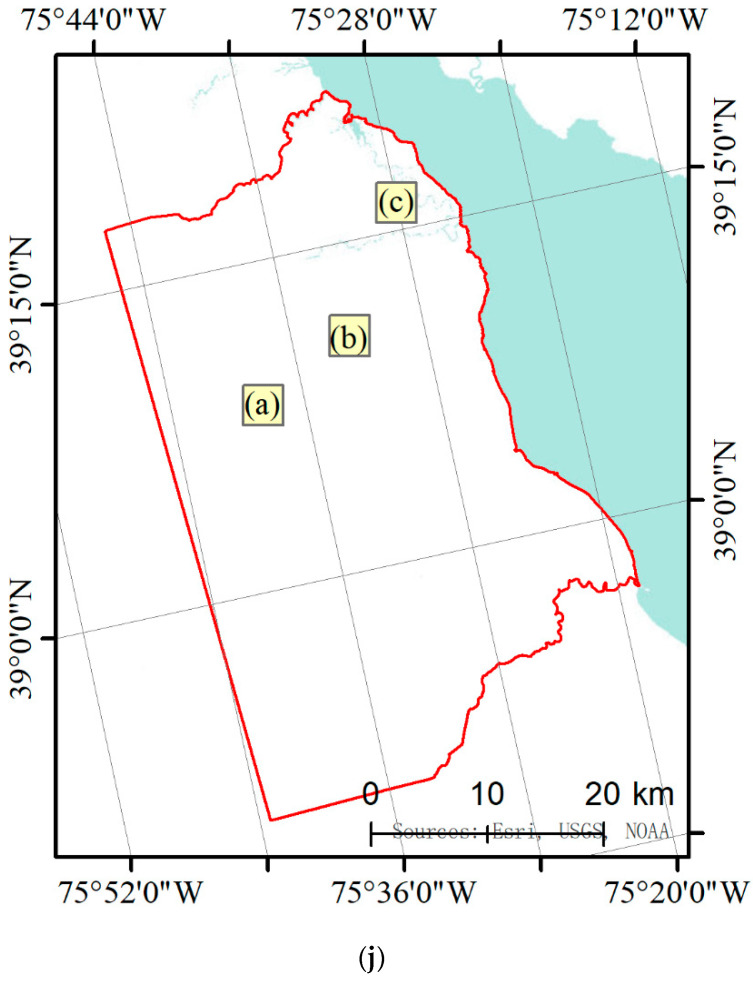
Details of the pre-classification land cover map versus the decision fusion land cover map. The three locations, shown at a larger scale, respectively contain (**a**) wetland and agriculture, (**b**) impervious and tree canopy, and (**c**) water and wetland, while (**d**–**f**) are the same regions with decision fusion results and (**g**–**i**) are the same regions with manually interpreted land cover. (**j**) is the location of the sample regions.

As shown in Figure 16a,d,g, the decision fusion result corrected some wetland regions that were misclassified as forest in the pre-classification process. In Figure 16b,e,h the pre-classification map and decision map are similar. Most tree canopy was misclassified as turf and the decision fusion method failed to correct them. Some turf patches misclassified as forest in the pre-classification were corrected in the decision map. In Figure 16c,f,i, the decision fusion method also failed to correct the large area of turf that was misclassified as agriculture in the pre-classification map. Moreover, the decision fusion method connected the areas of agriculture, which led to misclassification of a small region of wetland. The decision fusion method succeeded in correcting a small water area that was misclassified as agriculture in the pre-classification map. This experiment demonstrated that the decision fusion method tends to connect large patches that belong to the same class. This conforms to the first law of geography [57] but may also lead to misclassification.

**Figure 17:**
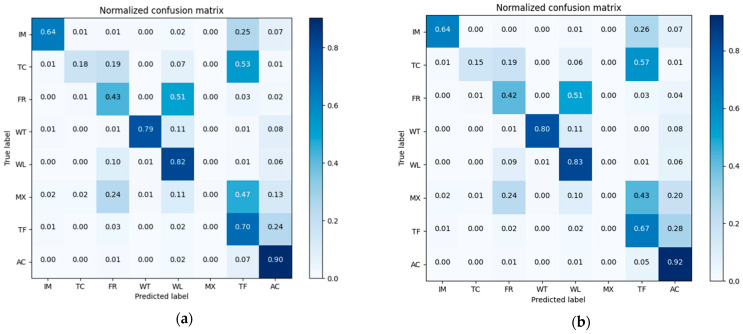
Confusion matrixes in Kent County for (**a**) pre-classification results and (**b**) the neighborhood spatial relationship and fuzzy classification fusion results.

**Figure 18:**
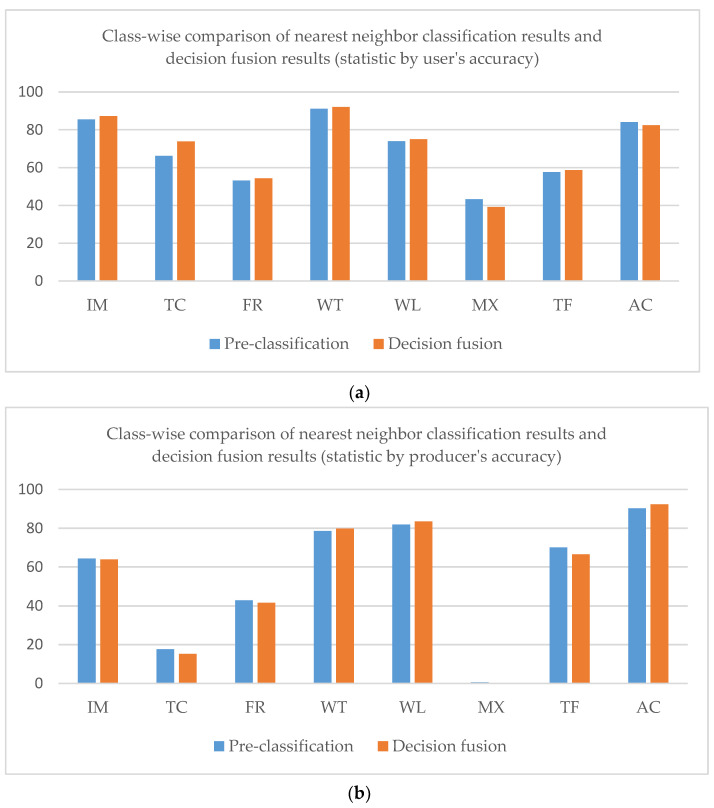
Class-wise comparison of fuzzy SVM classification results and decision fusion results (statistic by user’s accuracy (**a**) and producer’s accuracy (**b**)) in Kent County.

Normalized Confusion matrixes of classification results in Kent County is shown in Figure 17. The overall accuracy of the nearest neighbor classification result is 73.64%, while that for the decision fusion result is 74.26%. As to the Kappa coefficient, the nearest neighbor classification kappa coefficient is 0.648 and the decision fusion result is 0.653. Impervious surfaces misclassified as water were corrected in the decision result. As shown in Figure 17, tree canopy decreased in the decision result and more tree canopy was misclassified as turf in the decision result. Some forest cover was corrected from misclassification as turf, wetland, and impervious surfaces in the pre-classification but some of the forest patches were misclassified as agriculture in the decision fusion. Some misclassified mixed land in pre-classification was corrected to water in the decision fusion, and some misclassified mixed land in pre-classification was corrected to wetland in the decision fusion. No mixed land was corrected by the decision fusion process. Some turf that was correctly classified in pre-classification was then mis-labeled as tree canopy in the decision fusion result. A comparison of class-wise fuzzy SVM classification and decision fusion is shown in Figure 18.

As shown in Figure 18, the decision fusion user’s accuracy and producer’s accuracy did not change much compared with the fuzzy SVM classification result. We will analyze the reason for this result in the following section.

## 4. Discussion

### 4.1. Experiments Analysis

**Figure 19:**
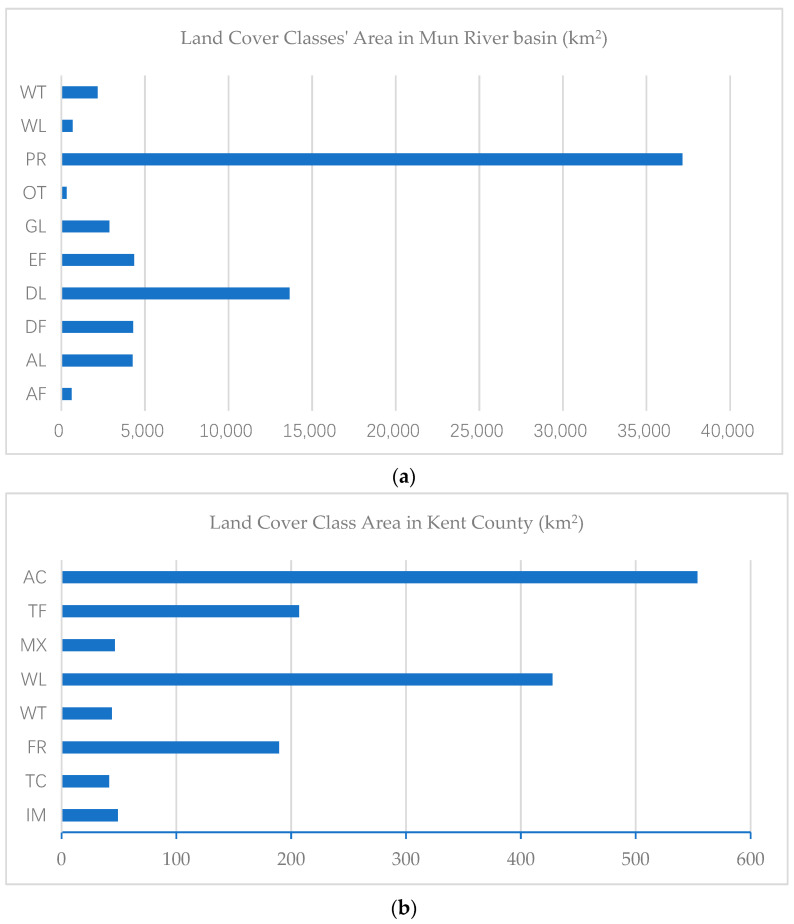
Area of land cover classes in the Mun River Basin (**a**) and Kent County (**b**). The area distribution of classes in the Mun River Basin is imbalanced compared with Kent County.

The first experiment’s overall accuracy improved 5.24%, whereas the second experiment’s overall accuracy only improved 0.62%. The main difference between the two regions is the distribution pattern of land cover classes. In the first region, the land cover distribution is imbalanced. As shown in Figure 19, the standard deviation of land cover classes in the Mun River Basin is 10,685.1, whereas the standard deviation of land cover classes in Kent County is 184.6.

**Figure 20:**
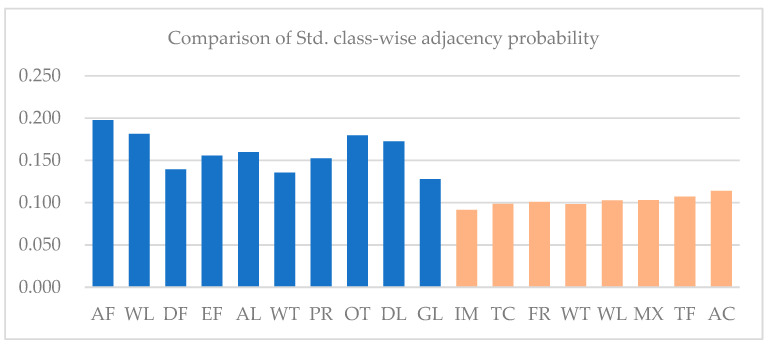
Comparison of standard deviation of class-wise adjacency probability in Kent County and the Mun River Basin.

The imbalance of the distribution of land cover types is also inferred from Table 1 and Table 2, the probability of land cover types being adjacent to each other in sample regions. As shown in Figure 20, class-wise standard deviations of the probability of adjacency in the Mun River Basin are all higher than Kent County, caused by the extensive distribution of paddy rice in the former leading to a high probability of other land cover adjacency to paddy rice. In contrast, land cover class distribution in Kent County is relatively balanced.

The imbalanced distribution of land cover classes meant a higher probability that classes with larger areas would have higher weights in the decision fusion process. This was also proved by the confusion matrix. More patches were misclassified as paddy rice in the decision fusion result. The problem was mainly caused by the single use of adjacency probability derived from the graph, which represents the global probability of adjacency rather than the local probability. Therefore, extracting the local probability of adjacency through more knowledge and spatial patterns is a worthwhile direction of study.

Kent County’s land cover distribution is relatively balanced. However, overall accuracy only improved 0.62%. There were 1632 among 52,719 objects that the decision fusion process successfully corrected from misclassification in pre-classification. The class membership of the corrected objects that were wrongly classified was close to the corrected class membership. The performance of pre-classification is vital in the performance of decision fusion.

There were 1395 among 52,719 objects that the pre-classification correctly classified but the decision fusion result misclassified. Focusing on these 1395 objects, two situations that led to incorrect classification were found, as shown in Table 3.

The first situation that caused misclassification is similar to the Mun River Basin. When the membership of two classes was close, so was that of the adjacent object. The probability of adjacency of the false class was higher, shown in Table 3 Situation 1, and the decision tended to classify them as classes with higher adjacency probability like B-B. The second situation was caused by the “siphonic effect”. Although membership of the two classes was not very close, the high membership of adjacent objects like Class B caused misclassification as well.

To sum up, two problems were found to cause misclassification in the decision process. First, the single use of global probability of adjacency caused the tendency of classification to those classes with larger areas. Second, the “siphonic effect” led to misclassification caused by high membership probability of neighborhood objects.

### 4.2. Segmentation Effects

**Figure 21:**
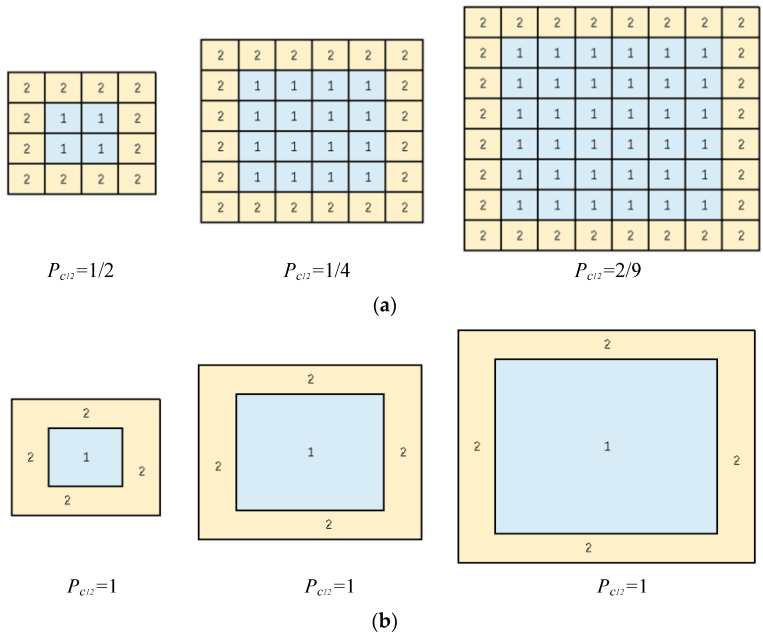
The object-oriented method for pre-classification was chosen based on whether the model uses (**a**) pixels for pre-classification or (**b**) objects for pre-classification. The object model is more robust compared to the pixel model, which makes the built graph more applicable to it.

The adjacency estimation will ulteriorly influence the fusion results. So that the results of the graph-based neighborhood adjacency estimation are depending on the seg-mentation result. The segmentation scale will influence adjacency estimation as shown in Figure 21.

In the experiment, we segmented the image used multi-resolution segmentation with a self-defined segment scale in the eCognition software. In the experiment, we set a proper segmentation scale as 60 in the first experiment and 80 in the second experiment. The adjacency possibility is calculated by the segmented polygons in the two-study case. However, the second experiment indicate that fusion result doesn’t improve much even when the adjacency possibility is calculated in the same scale with the object-oriented classification process. In The second experiment, pre-classification overall accuracy is 73.64%. Much improvement is hardly achievable by incorporating adjacency possibilities. These results indicate that the fusion results are hardly affected by segmentation scale provided that proper segmentation scale is set in the object-oriented classification process.

## 5. Conclusions

In this paper, we presented a method to extract the probability of adjacency between classes using graph theory. In order to utilize adjacency probability in the decision fusion process, the pre-fuzzy-classification results and the adjacency probability were combined at the decision level. Two experiments exhibited improvement in the overall accuracy. Al though the overall accuracies are not significantly improved. As for the class-wise accuracy, the user’s accuracies are obviously improved in term of most of the classes.

In the first study case, Landsat data are used for pre-classification and the overall accuracy is 61.71%. It is likely to achieve more improvement in the fusion result compared with the second study case, which pre-classification overall accuracy is 73.64%. And indeed, the first study case achieved the 5.2% overall accuracy improvement and the second study case only improved by 0.6%. Another reason may lead to the limited improvement of the overall accuracy in the second study is that we are using a simulated “true” map rather than a manually interpreted map as in the first study case. It causes inaccuracy of adjacency possibility in the second study case. Although the overall accuracy of the decision fusion result is unsatisfactory in the second study case, user’s accuracy is obviously improved. We only used the 1th order adjacency degree to dig the adjacency information. Future work that explores more information from the graph is needed for overall accuracy improvement.

We analyzed the results and found two major problems in the proposed method. First, using adjacency probability derived from the samples of the regions to be classified resulted in imbalanced classification. Second, directly multiplying the adjacency probability and membership caused neighborhood objects to experience the “siphonic effect”.

There are improvements feasible to further digging the adjacency knowledge using graph theory. In this paper, we simply used the first order adjacent degree in the sampled graph to inference the adjacency probability. There was second order and even third order adjacency in the graph theory. Also, graph embedding theory and graph neural network theory were employed to extract spatial relationships between classes.

Researchers have used knowledge to inference land cover classes. The general scheme is to first match the scene knowledge, then use that knowledge to classify the im-age again. This is a practical effort. In our research, we found that using neighborhood adjacency probability caused problems. A practical improvement might be to use scene knowledge, inferencing adjacency probability from the scene knowledge rather than statistics from the whole map. Another direction is to embed the knowledge into the classifier. Some researchers have already used a GNN (Graph Neural Network) model to classify remote sensing imagery [39,40]. These studies have proved that the spatial relationship introduced by GCN boosts the performance and robustness of the classification model. Application of graph theory in extracting spatial relationship information and the use of prior knowledge in remote sensing image classification is worth further research in the future.

## Figures and Tables

**Table 1:** Probability of land cover types being adjacent to each other in the Mun River Basin.

	AF ^1^	WL ^2^	DF ^3^	EF ^4^	AL ^5^	WT ^6^	PR ^7^	OT ^8^	DL ^9^	GL ^10^
AF	0.659	0.001	0.000	0.000	0.000	0.001	0.000	0.000	0.003	0.000
WL	0.009	0.613	0.004	0.004	0.004	0.038	0.007	0.013	0.003	0.004
DF	0.007	0.017	0.489	0.001	0.020	0.028	0.046	0.029	0.042	0.060
EF	0.000	0.004	0.000	0.524	0.008	0.003	0.010	0.006	0.011	0.010
AL	0.000	0.021	0.027	0.046	0.566	0.054	0.077	0.045	0.037	0.032
WT	0.025	0.130	0.025	0.012	0.032	0.478	0.055	0.022	0.014	0.043
PR	0.037	0.162	0.263	0.225	0.279	0.304	0.661	0.196	0.196	0.258
OT	0.000	0.003	0.002	0.001	0.002	0.001	0.002	0.600	0.001	0.001
DL	0.258	0.026	0.124	0.136	0.062	0.036	0.087	0.057	0.635	0.128
GL	0.006	0.022	0.065	0.051	0.028	0.057	0.055	0.032	0.058	0.463

^1^ artificial forest; ^2^ wet land; ^3^ deciduous forest; ^4^ evergreen forest; ^5^ developed land; ^6^ water; ^7^ paddy rice; ^8^ others; ^9^ dry land; ^10^ grassland.

**Table 2:** Probability of land cover types being adjacent to each other in Kent County.

	IM ^1^	TC ^2^	FR ^3^	WT ^4^	WL ^5^	MX ^6^	TF ^7^	AC ^8^
IM	0.101	0.027	0.144	0.018	0.183	0.048	0.170	0.308
TC	0.057	0.045	0.173	0.024	0.155	0.041	0.169	0.335
FR	0.051	0.028	0.175	0.033	0.182	0.039	0.159	0.334
WT	0.036	0.022	0.193	0.028	0.215	0.046	0.165	0.295
WL	0.050	0.022	0.150	0.030	0.216	0.049	0.149	0.334
MX	0.058	0.022	0.127	0.026	0.206	0.058	0.160	0.343
TF	0.055	0.028	0.145	0.027	0.170	0.047	0.162	0.366
AC	0.046	0.024	0.142	0.022	0.176	0.045	0.163	0.382

^1^ impervious; ^2^ tree canopy; ^3^ forest; ^4^ water; ^5^ wetland; ^6^ mixed; ^7^ turf; ^8^ agriculture.

**Table 3:** Two situations demonstrate the objects misclassified by decision fusion. Problems causing the misclassification are marked in underline.

**Situation 1:**					
**Object Membership**	**Adjacent Membership**	**Adjacency Probability**	**True Class**	**Pre-Classified Result**	**Decision Result**
Class A: 0.35	Class A: 0.37	A-A: 0.1 B-B: 0.3 A-B:0.05	Class A	Class A	Class B
Class B: 0.32	Class B: 0.23				
**Situation 2:**					
**Object Membership**	**Adjacent Membership**	**Adjacency Probability**	**True Class**	**Pre-Classified Result**	**Decision Result**
Class A: 0.35	Class A: 0.27	A-A: 0.1 B-B: 0.3 A-B:0.05	Class A	Class A	Class B
Class B: 0.22	Class B: 0.73				

## Data Availability

The data presented in this study are openly available in [Mendeley Data] at [DOI:10.17632/2sgkcpfh65.2], accessed on 13 August 2021.
